# Accelerometer-derived “weekend warrior” physical activity pattern and risk of age-related eye diseases: a prospective cohort study

**DOI:** 10.1186/s40662-026-00480-6

**Published:** 2026-03-10

**Authors:** Yuze Mi, Jiahui Zong, Shangdong Wang, Qinnan Zhu, Shaokai Lin, Xinni Zheng, Yanggang Hong, Jiawei Zhou, Liang Ye

**Affiliations:** 1 State Key Laboratory of Eye Health, Eye Hospital and National Engineering Research Center of Ophthalmology and Optometry, Wenzhou Medical University, Wenzhou, China; 2 The Third Affiliated Hospital of Wenzhou Medical University (Ruian People’s Hospital), Wenzhou, China; 3 Wenzhou Medical University, Wenzhou, China; 4 School of Ophthalmology and Optometry and Eye Hospital, Wenzhou Medical University, 270 Xueyuan West Road, Wenzhou, 325027 China

**Keywords:** Physical activity, Weekend warrior, Cataract, Glaucoma, Diabetic retinopathy, Age-related macular degeneration, UK Biobank

## Abstract

**Background:**

International guidelines recommend at least 150 min of weekly moderate-to-vigorous physical activity (MVPA), but whether concentrated versus distributed activity patterns differ in their associations with age-related eye diseases remains unclear.

**Methods:**

This prospective cohort study included 86,271 UK Biobank participants free of age-related eye diseases at baseline. Physical activity was assessed using wrist-mounted triaxial accelerometers (Axivity AX3) over seven consecutive days. Two MVPA thresholds were examined: ≥ 150 min/week (primary) and ≥ 300 min/week (secondary). Participants were categorized as: inactive (below threshold), weekend warriors (WW; meeting threshold with ≥ 50% MVPA concentrated within 1–2 days), or regularly active (meeting threshold without WW criteria). Cox proportional hazards regression models were used to assess associations over a median follow-up of 7.9 years.

**Results:**

At the ≥ 150 min/week threshold, both WW (hazard ratio [HR] = 0.89, 95% confidence interval [CI]: 0.84–0.94, *P* < 0.001) and regularly active patterns (HR = 0.93, 95% CI: 0.87–0.99, *P* = 0.028) were associated with a reduced risk of cataract compared to inactivity. The WW pattern was also associated with a reduced risk of diabetic retinopathy (DR, HR = 0.74, 95% CI: 0.55–0.99, *P* = 0.043) and age-related macular degeneration (AMD, HR = 0.85, 95% CI: 0.75–0.97, *P* = 0.016). Direct comparisons between the WW and regularly active patterns showed no significant differences for these conditions (all *P* > 0.05), except for a nominal difference in glaucoma (*P* = 0.036). At the ≥ 300 min/week threshold, only the WW pattern remained significantly associated with reduced risk of cataract (HR = 0.91, 95% CI: 0.86–0.97, *P* = 0.002) and glaucoma (HR = 0.86, 95% CI: 0.75–0.97, *P* = 0.019).

**Conclusion:**

Both the WW and regularly active patterns demonstrate protective associations with age-related eye diseases compared to inactivity, with no statistically significant differences between the two active groups for most outcomes. These findings suggest that the WW approach is a viable and flexible alternative for individuals who find it difficult to maintain daily physical activity.

**Supplementary Information:**

The online version contains supplementary material available at 10.1186/s40662-026-00480-6.

## Background

Age-related eye diseases, including cataract, age-related macular degeneration (AMD), diabetic retinopathy (DR), and glaucoma, represent leading causes of preventable blindness among the older population worldwide [1]. As of 2020, there were an estimated 23.8 million individuals aged 50 years or older who were blind [1]. These conditions not only significantly impair individuals’ quality of life, but also impose substantial economic burdens on healthcare systems [2]. Age-related eye diseases result from a complex interplay among genetic predisposition, environmental influences, and modifiable behavioral factors. While genetic factors are largely uncontrollable, and environmental changes are costly, modifying behavioral factors offers a more accessible and cost-effective pathway to prevent or delay the onset and progression of age-related eye diseases [3].

Physical activity is one of the most recognized behavioral factors, and substantial evidence demonstrates its beneficial roles against various age-related eye diseases [4–7]. Physical activity can enhance ocular blood flow, reduce oxidative stress, and improve insulin sensitivity, contributing to better ocular health [8]. Current guidelines recommend that adults engage in moderate-to-vigorous physical activity (MVPA) for a minimum of 150 min per week for health benefits, and exceeding 300 min of MVPA per week can provide additional benefits [9, 10]. However, it remains challenging for working people to keep a regular exercise schedule during the week due to the demands of a fast-paced society. Therefore, a new exercise model named the “weekend warrior” (WW) has been proposed, which offers a practical alternative by consolidating physical activity into one or two days, such as the weekends [11].

The WW pattern offers a potentially convenient approach that enables individuals to accrue recommended weekly physical activity within a compressed timeframe. For busy individuals, this approach provides a flexible alternative to navigate modern lifestyle constraints while striving to meet recommended guidelines. Given the rising popularity of the WW pattern in contemporary society, investigating the health benefits associated with this exercise modality has become imperative [12]. The latest evidence from large-scale prospective cohort studies indicates that the WW pattern provides comparable protection as the regular active pattern against a broad spectrum of chronic diseases, including cardiovascular events [11], type 2 diabetes [13], and neurodegenerative diseases [14, 15]. This provides a strong basis for exploring the potential impact of the WW pattern on age-related eye diseases, given their clinical co-morbidity and shared risk factors.

To date, the association between WW patterns and age-related eye diseases remains underexplored, with prior studies limited by small sample sizes and self-reported data susceptible to recall bias. Based on evidence that the WW pattern provides comparable cardiometabolic benefits to regularly active patterns, we hypothesized that: (1) Primary hypothesis: Both the WW and regularly active patterns are associated with a significantly reduced risk of age-related eye diseases compared to physical inactivity. (2) Secondary hypothesis: Given the shared pathophysiological mechanisms (e.g., oxidative stress, chronic inflammation, vascular dysfunction) between cardiometabolic conditions and ocular diseases, the WW pattern demonstrates protective associations comparable to those of regular activity. (3) Exploratory hypothesis: These associations may vary significantly across different demographic subgroups, particularly according to age and sex.

## Methods

### Ethics approval

The study was approved by the North-West Multicenter Research Ethics Committee (Reference No. 06/MRE08/65) in accordance with the Declaration of Helsinki, with all participants providing informed consent via electronic signatures. The present study, conducted under application number 302523, follows the Strengthening the Reporting of Observational Studies in Epidemiology (STROBE) guidelines for cohort studies. Additional details are available at https://www.ukbiobank.ac.uk/.

### Data sources and study population

The UK Biobank is a large prospective cohort study that recruited 502,629 participants aged 40–69 years from 22 assessment centers across the UK between 2006 and 2010, with ongoing follow-up [16]. Between February 2013 and December 2015, UK Biobank participants who had provided a valid email address were invited via email to participate in a physical activity monitoring sub-study. Participants were requested to wear an accelerometer for seven consecutive days. A total of 103,662 participants provided continuous 7-day physical activity data collected using a wrist-worn triaxial accelerometer (Axivity AX3; Axivity Ltd., Newcastle upon Tyne, UK). To ensure analytical consistency, only participants with a complete 7-day wear duration were included in the final study cohort [17]. The device recorded acceleration at 100 Hz within a ± 8 g dynamic range, with gravity-calibrated signals processed into 5-s epochs representing mean vector magnitudes. Non-wear periods were identified as continuous intervals of ≥ 60 min with standard deviation < 13.0 mg across all three axes. The Axivity AX3 has demonstrated comparable performance to GENEActiv accelerometers utilized in other large-scale cohort studies [18].

To ensure data integrity and analytical rigor, systematic baseline exclusion criteria were applied. Participants were excluded for the following reasons: (1) inadequate accelerometer wear duration (< 7 complete days); (2) missing monitoring data during any hourly interval within the 24-h cycle; (3) calibration discrepancies exceeding 10 mg or excessive acceleration measurements (> 100 mg); (4) incomplete covariate information; (5) insufficient follow-up duration (< 3 years); or (6) prevalent age-related eye diseases (cataract, glaucoma, DR, or AMD) at baseline. Of the 103,662 participants who provided accelerometer-based physical activity data over a 7-day period, 91,948 met criteria (1) to (5). After excluding 5,677 participants with prevalent cataract, glaucoma, DR, or AMD at baseline, 86,271 were included in the final analysis.

### Classification of physical activity patterns

Physical activity behaviors were classified using a validated machine learning framework specifically developed for the UK Biobank [17, 19]. A Random Forest classifier was employed to identify activity states in 5-s epochs. This model was trained on a free-living ground-truth dataset (annotated via wearable cameras), achieving an overall accuracy of 88% and a Cohen’s *κ* of 0.80 for distinguishing between MVPA, light activity, sedentary behavior, and sleep [17, 19]. Rather than relying on a simple static acceleration threshold, our machine learning model identifies MVPA by analyzing spectral and frequency-domain features of the 100 Hz triaxial signal [17]. This classification boundary corresponds to a metabolic equivalent (MET) ≥ 3 [17, 19] and aligns closely with the established hardware-calibrated threshold of approximately 100 mg (milli-gravities) for moderate-intensity activity [17, 20]. Weekly MVPA was calculated by determining the daily fraction of time spent in MVPA (minutes/1440) and aggregating these values across the 7-day period [11]. Our categorization framework incorporated established guidelines from leading public health authorities, specifying ≥ 150 weekly minutes for basic health benefits and ≥ 300 weekly minutes for additional advantages [21, 22].

Participants were classified into three groups: the inactive group (< 150/300 min/week), the WW group (≥ 150/300 min/week with ≥ 50% of total MVPA concentrated within 1–2 days), and the regularly active group (≥ 150/300 min/week but not meeting WW criteria). Following established protocols, the WW pattern was operationally defined as accumulating ≥ 50% of total weekly MVPA within 1–2 days. It is important to note that these active days could occur on any day of the week and were not restricted to Saturday and Sunday. This definition aims to capture the physiological effect of concentrated versus distributed activity volumes, regardless of the specific calendar days on which the activity occurred.

### Age-related eye diseases

Age-related eye diseases were identified using International Classification of Diseases (ICD)-10 diagnostic codes from linked National Health Service electronic healthcare records, including hospital inpatient data (Hospital Episode Statistics for England, Scottish Morbidity Record for Scotland, and Patient Episode Database for Wales), primary care data from general practitioners, and death records. This approach has been validated in previous UK Biobank ophthalmic research [23–25].

The analysis focused on four conditions: cataract, glaucoma, DR, and AMD (diagnostic codes detailed in Table S1). Participants were prospectively followed from the date of accelerometer assessment completion until the occurrence of the primary endpoint, defined as the first recorded diagnosis of the outcome of interest (i.e., incident cataract, glaucoma, DR, or AMD). For individuals who did not develop these conditions, data were censored at the earliest of the following: (1) death from any cause, (2) withdrawal from the UK Biobank study, or (3) the administrative end of follow-up (October 31, 2022, for England; August 31, 2022, for Scotland; and May 31, 2018, for Wales).

### Covariates

Participant age was calculated as the interval between birth date and accelerometer cessation date. Self-reported demographic characteristics included ethnicity, which was classified into the predefined UK Biobank categories: White, Asian, Black, or Mixed/Other. This classification aligns with standard UK demographic reporting, where ‘Hispanic’ is not categorized as an independent ethnicity. Biological sex was recorded as male or female. Socioeconomic status was assessed using the Townsend deprivation index. Educational attainment was quantified as the age at completion of full-time education; for participants reporting only degree or qualification status, equivalent completion ages were assigned according to established protocols [26].

Employment status was classified as either paid employment/self-employment or retired/unemployed. Smoking and alcohol consumption patterns were categorized as current, former, or never use. Anthropometric measurements, including weight and height, were obtained by trained nursing staff and used to calculate the body mass index (BMI) in kilograms per square meter (kg/m^2^). Prevalent diabetes and hypertension were defined as binary variables (yes/no) based on baseline self-reported physician diagnoses and concurrent use of relevant medications (antihypertensive or antidiabetic agents, Table S2).

### Statistical analysis

Baseline characteristics of participants were summarized across different physical activity patterns using means and standard deviations for continuous variables and frequencies with proportions for categorical variables. Standardized mean differences (SMD) were calculated to quantify the magnitude of between-group differences. Median follow-up duration was estimated using the reverse Kaplan–Meier method. In this procedure, the status indicator is flipped: individuals who remained event-free until the end of the study are treated as having reached the ‘event’ (completed follow-up), while those who experienced the primary endpoint (e.g., a cataract diagnosis) or died are treated as censored at the time of that event. This approach ensures that the estimated median follow-up time reflects the full duration for which participants were at risk and under observation, rather than being biased downward by the occurrence of early events.

The associations between different physical activity patterns and incident age-related eye diseases were examined using multivariable Cox proportional hazards regression models, adjusting for all covariates. The proportional hazards assumption was verified through Schoenfeld residual testing. To minimize the risk of reverse causality, subclinical or undiagnosed eye disease might reduce physical activity levels prior to formal diagnosis—we implemented a landmark analysis approach. In our primary analysis, we excluded participants who experienced an outcome within the first 0.5 years of follow-up. Furthermore, we conducted a sensitivity analysis excluding the first 3 years of follow-up. This longer washout period helps ensure that the observed associations are not driven by pre-existing conditions that may have influenced baseline activity levels. Hazard ratios (HRs) for cataract, glaucoma, DR, and AMD were compared across three physical activity groups (WW, regularly active, and inactive) using MVPA thresholds of 150 and 300 min per week, respectively. To enable direct comparison between active patterns, additional analyses were conducted using the regularly active group as the reference category. Kaplan–Meier survival curves were constructed to visualize time-to-event outcomes across the three activity categories. Restricted cubic spline analyses with 3 degrees of freedom were performed to examine potential non-linear dose–response relationships between continuous weekly MVPA and each outcome, with 150 min/week as the reference.

The following sensitivity analyses were conducted to assess the robustness of primary findings: (1) To explore potential effect modification, subgroup analyses were performed by sex and age. For age-stratified analyses, the cohort was dichotomized into two groups (< 62 vs. ≥ 62 years) based on the median age of the study population to ensure balanced group sizes and optimized statistical power; (2) Missing covariate data were addressed using multiple imputation by chained equations (MICE) with the predictive mean matching algorithm to generate 10 imputed datasets, incorporating all study covariates and survival outcomes to preserve statistical associations [27]; (3) To further mitigate reverse causality from undiagnosed conditions, analyses were repeated after excluding the initial 3 years of follow-up; (4) Fine-Gray subdistribution hazard models were employed to account for the competing risk of death [28]; (5) Sensitivity analyses using alternative WW concentration thresholds (≥ 40% and ≥ 60% of weekly MVPA in 1–2 days) were conducted to test the robustness of WW classification; and (6) Subtype-specific analyses were conducted for primary open-angle glaucoma (POAG, ICD-10: H40.1), primary angle-closure glaucoma (PACG, H40.2), and non-PACG glaucoma using the same covariate adjustments.

Given the multiple comparisons across 4 outcomes × 2 activity patterns × 2 thresholds, Bonferroni correction was applied for primary analyses (corrected *α* = 0.003). Stratified and interaction analyses were considered exploratory and hypothesis-generating. All analyses were performed using R software (version 4.3.3), with statistical significance defined as *P* < 0.05 (two-sided) for primary analyses.

## Results

### Baseline characteristics

The study cohort included 86,271 participants with a median age of 62.6 years, of whom 56.5% were female (Table 1). Using the recommended guideline of 150 min weekly MVPA, the participants were divided into three categories: 37,486 participants (43.5%) classified as WWs, 20,022 (23.2%) as regularly active, and 28,763 (33.3%) as inactive. When applying the 300 min weekly MVPA criterion, the distribution shifted to 18,336 WWs (21.3%), 15,499 regularly active participants (18.0%), and 52,436 inactive individuals (60.8%). Compared with inactive participants, the WW group was younger (median: 62.6 vs. 63.7 years, SMD = 0.12), had a higher proportion of males (48.7% vs. 32.9%, SMD = 0.33), and showed slightly higher socioeconomic status (median Townsend index: − 2.6 vs. − 2.5, SMD = 0.05). Furthermore, the WW group exhibited higher employment rates (62.5% vs. 57.0%, SMD = 0.11), healthier lifestyle profiles (e.g., lower smoking prevalence: 5.5% vs. 8.6%, SMD = 0.13), lower BMI (median: 25.7 vs. 27.2 kg/m^2^, SMD = 0.40), and lower prevalence of hypertension (21.9% vs. 29.9%, SMD = 0.18) and diabetes (2.3% vs. 5.1%, SMD = 0.14). All between-group differences were statistically significant (*P* < 0.001). Participants in the regularly active category generally demonstrated intermediate characteristics between the inactive and WW groups (Table 1).

**Table 1:** Characteristics of participants by different thresholds of MVPA per week

Patient characteristics	≥ 150 min of MVPA per week			≥ 300 min of MVPA per week		
	Inactive^a^ (n = 28,763)	Regular^a^ (n = 20,022)	WW^a^ (n = 37,486)	Inactive^b^ (n = 52,436)	Regular^b^ (n = 15,499)	WW^b^ (n = 18,336)
Demographic and socioeconomic characteristics						
Age (years)	63.7 [56.4; 68.7]	61.1 [53.9; 66.8]	62.6 [55.6; 67.6]	63.2 [56.0; 68.2]	60.9 [53.8; 66.5]	62.4 [55.4; 67.4]
Sex, n (%)						
Female	19,314 (67.1)	10,192 (50.9)	19,219 (51.3)	33,255 (63.4)	7,426 (47.9)	8,044 (43.9)
Male	9,449 (32.9)	9,830 (49.1)	18,267 (48.7)	19,181 (36.6)	8,073 (52.1)	10,292 (56.1)
Townsend deprivation index	− 2.5 [− 3.8; − 0.3]	− 2.1 [− 3.6; 0.5]	− 2.6 [− 3.9; − 0.5]	− 2.5 [− 3.8; − 0.3]	− 2.1 [− 3.6; 0.5]	− 2.7 [− 3.9; − 0.6]
Ethnic type, n (%)						
White	27,798 (96.6)	19,352 (96.7)	36,540 (97.5)	50,725 (96.7)	15,013 (96.9)	17,952 (97.9)
Black	290 (1.0)	166 (0.8)	256 (0.7)	504 (1.0)	116 (0.7)	92 (0.5)
Asian	384 (1.3)	229 (1.1)	334 (0.9)	652 (1.2)	170 (1.1)	125 (0.7)
Other	291 (1.0)	275 (1.4)	356 (0.9)	555 (1.1)	200 (1.3)	167 (0.9)
Educational attainment (years)	18.0 [16.0; 20.0]	20.0 [16.0; 20.0]	20.0 [16.0; 20.0]	18.0 [16.0; 20.0]	20.0 [17.0; 20.0]	20.0 [16.0; 20.0]
Employment status, n (%)						
Paid employment or self-employed	16,399 (57.0)	13,438 (67.1)	23,445 (62.5)	31,244 (59.6)	10,497 (67.7)	11,541 (62.9)
Retired or unemployed	12,364 (43.0)	6584 (32.9)	14,041 (37.5)	21,192 (40.4)	5002 (32.3)	6795 (37.1)
Lifestyle characteristics						
Smoking status, n (%)						
Never	15,860 (55.1)	11,582 (57.8)	22,180 (59.2)	29,795 (56.8)	8945 (57.7)	10,882 (59.3)
Previous	10,421 (36.2)	7216 (36.0)	13,233 (35.3)	18,724 (35.7)	5643 (36.4)	6503 (35.5)
Current	2482 (8.6)	1224 (6.1)	2073 (5.5)	3917 (7.5)	911 (5.9)	951 (5.2)
Alcohol status, n (%)						
Never	1129 (3.9)	494 (2.5)	841 (2.2)	1755 (3.3)	352 (2.3)	357 (1.9)
Previous	957 (3.3)	530 (2.6)	857 (2.3)	1543 (2.9)	405 (2.6)	396 (2.2)
Current	26,677 (92.7)	18,998 (94.9)	35,788 (95.5)	49,138 (93.7)	14,742 (95.1)	17,583 (95.9)
Clinical characteristics						
Body mass index (kg/m^2^)	27.2 [24.4; 30.7]	25.3 [23.0; 27.9]	25.7 [23.4; 28.3]	26.6 [24.0; 29.8]	25.1 [22.9; 27.6]	25.4 [23.3; 27.9]
Hypertension, n (%)	8588 (29.9)	4098 (20.5)	8197 (21.9)	14,163 (27.0)	3014 (19.4)	3706 (20.2)
Diabetes, n (%)	1460 (5.1)	492 (2.5)	880 (2.3)	2134 (4.1)	334 (2.2)	364 (2.0)

*MVPA* = moderate-to-vigorous physical activity; *WW* = weekend warrior

Data are n (%) or median (quartile)

^a^Individuals classified as: inactive (below 150 min MVPA threshold), regular exercisers (≥ 150 min MVPA, not WW), weekend warrior (≥ 150 min MVPA with ≥ 50% over 1–2 days)

^b^Individuals classified as: inactive (below 300 min MVPA threshold), regular exercisers (≥ 300 min MVPA, not WW), weekend warrior (≥ 300 min MVPA with ≥ 50% over 1–2 days)

### Physical activity patterns and risks of age-related eye diseases

**Fig. 1:**
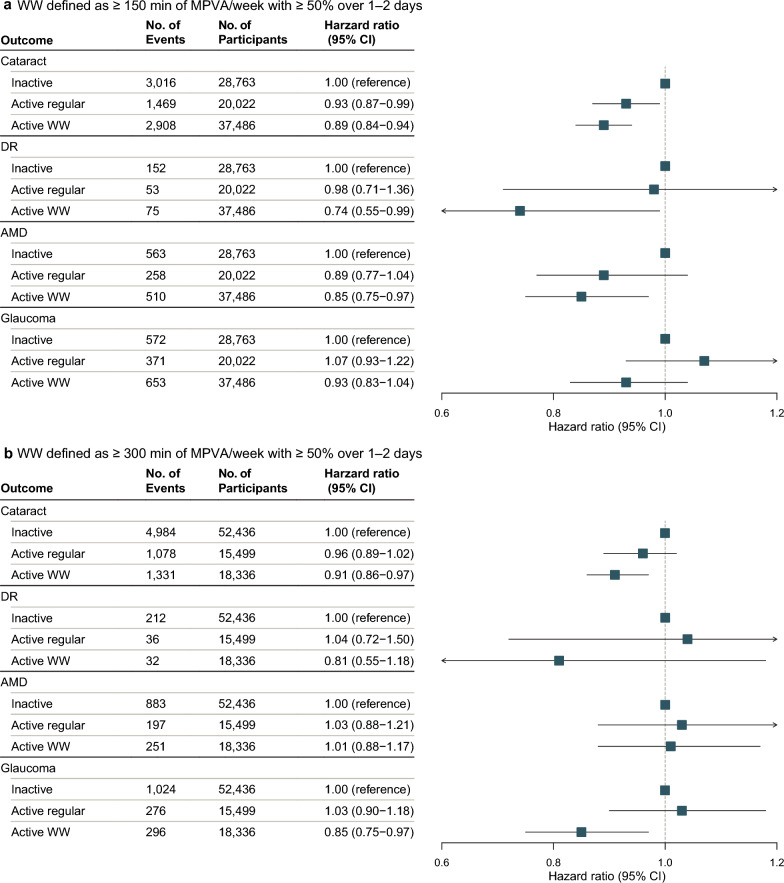
Multivariable-adjusted associations between physical activity pattern and incidence of age-related eye diseases. Forest plots display hazard ratios with 95% confidence intervals; the inactive group served as reference. **a** ≥ 150 min/week MVPA threshold. **b** ≥ 300 min/week MVPA threshold. MVPA, moderate-to-vigorous physical activity; AMD, age-related macular degeneration; DR, diabetic retinopathy; WW, weekend warrior

**Fig. 2:**
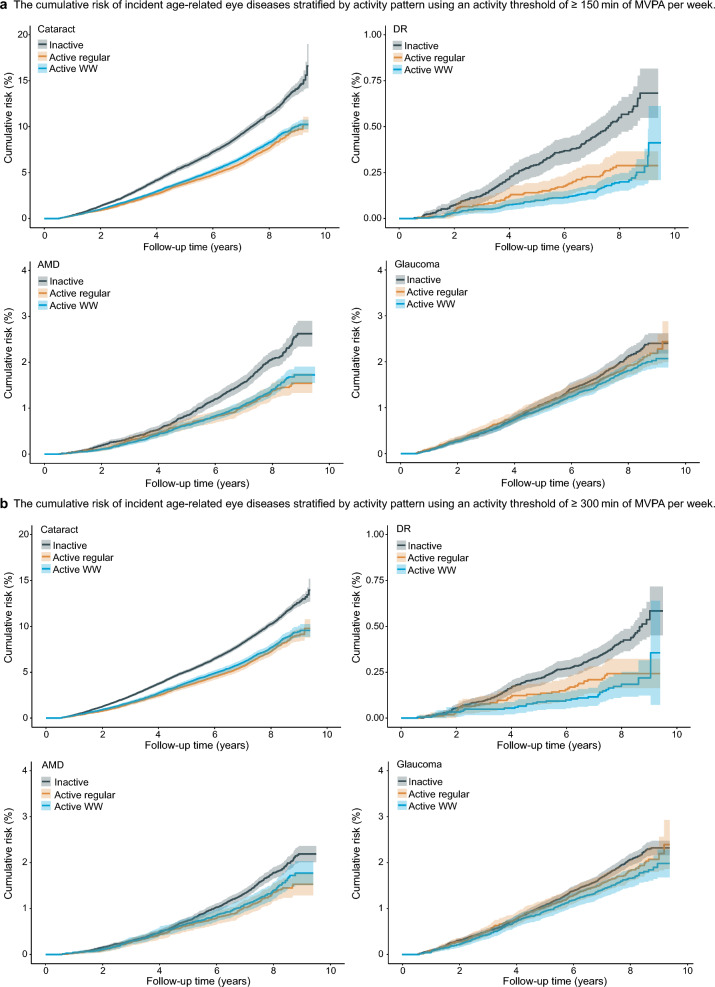
Cumulative incidence of age-related eye diseases stratified by physical activity pattern over a 10-year follow-up period. **a** ≥ 150 min/week MVPA threshold. **b** ≥ 300 min/week MVPA threshold. MVPA, moderate-to-vigorous physical activity; AMD, age-related macular degeneration; DR, diabetic retinopathy; WW, weekend warrior

During a median follow-up of 7.9 years, the incidence rates of glaucoma, DR, AMD, and cataract were 2.38 (95% CI: 2.27–2.50), 0.41 (95% CI: 0.37–0.47), 1.98 (95% CI: 1.88–2.09), and 11.32 (95% CI: 11.07–11.58) per 1000 person-years, respectively. Figures 1 and 2 show the associations between different physical activity patterns and different age-related eye diseases in the multivariable-adjusted models. At the ≥ 150 min/week threshold, the WW pattern was associated with reduced risk of cataract (HR = 0.89, 95% CI: 0.84–0.94, *P* < 0.001), DR (HR = 0.74, 95% CI: 0.55–0.99, *P* = 0.043), and AMD (HR = 0.85, 95% CI: 0.75–0.97, *P* = 0.016), while showing a non-significant trend toward reduced glaucoma risk (HR = 0.93, 95% CI: 0.83–1.04, *P* = 0.192). The regularly active pattern was associated with reduced cataract risk (HR = 0.93, 95% CI: 0.87–0.99, *P* = 0.028), while showing non-significant associations with DR (HR = 0.98, 95% CI: 0.71–1.36, *P* = 0.909), AMD (HR = 0.89, 95% CI: 0.77–1.04, *P* = 0.148), and glaucoma (HR = 1.07, 95% CI: 0.93–1.22, *P* = 0.359). Direct comparisons between WW and regularly active patterns showed no significant differences for these conditions (all *P* > 0.05), except for a nominal difference in glaucoma (*P* = 0.036; Table 2).

**Table 2:** Direct comparison of weekend warrior vs. regularly active patterns

Outcome	Threshold	N events (WW/Regular)	HR (95% CI)	*P* value
Cataract	≥ 150 min/week	2908/1469	0.96 (0.90–1.02)	0.219
	≥ 300 min/week	1331/1078	0.95 (0.88–1.03)	0.234
Diabetic retinopathy	≥ 150 min/week	75/53	0.75 (0.53–1.07)	0.111
	≥ 300 min/week	32/36	0.78 (0.48–1.25)	0.297
Age-related macular degeneration	≥ 150 min/week	510/258	0.96 (0.82–1.11)	0.550
	≥ 300 min/week	251/197	0.98 (0.82–1.19)	0.869
Glaucoma	≥ 150 min/week	653/371	0.87 (0.77–0.99)	0.036
	≥ 300 min/week	296/276	0.83 (0.70–0.98)	0.027

All models were adjusted for age, sex, Townsend deprivation index, ethnicity, education, employment status, smoking status, alcohol consumption, body mass index, hypertension, and diabetes

*HR* = hazard ratio; *CI* = confidence interval; *WW* = weekend warrior

When the threshold was increased to ≥ 300 min/week, only the WW pattern remained associated with reduced risk of cataract (HR = 0.91, 95% CI: 0.86–0.97, *P* = 0.002) and glaucoma (HR = 0.86, 95% CI: 0.75–0.97, *P* = 0.019), while showing non-significant associations with DR (HR = 0.80, 95% CI: 0.54–1.19, *P* = 0.270) and AMD (HR = 0.96, 95% CI: 0.81–1.15, *P* = 0.673). In contrast, the regularly active pattern showed no significant associations with any age-related eye disease at this higher threshold (cataract: HR = 0.96, 95% CI: 0.89–1.03, *P* = 0.232; DR: HR = 1.02, 95% CI: 0.68–1.55, *P* = 0.912; AMD: HR = 0.99, 95% CI: 0.82–1.19, *P* = 0.905; glaucoma: HR = 1.03, 95% CI: 0.90–1.18, *P* = 0.702). Direct comparisons at this threshold again showed no significant differences except for glaucoma (*P* = 0.027; Table 2, Figs. 1 and 2).

### Dose–response associations (RCS analysis)

Restricted cubic spline analyses (Figure S1) revealed significant non-linear associations for cataract (*P* for non-linearity = 0.007) and AMD (*P* = 0.001). For cataract, the risk decreased steeply up to approximately 200 min/week and then plateaued. For AMD, the lowest risk was observed around 150–200 min/week, with a slight attenuation of benefit at higher activity levels. In contrast, DR (*P* = 0.055) and glaucoma (*P* = 0.871) showed no significant deviation from linearity.

### Sensitivity analyses

The associations remained consistent after employing multiple imputation to account for missing covariate data, with no significant differences observed between the imputed and non-imputed datasets (Table S3, Figures S2 and S3). Further interaction tests revealed no significant effect modification by sex or age for the associations between physical activity patterns and cataract, DR, and AMD (all *P* values for interaction > 0.05).

Subtype-specific analyses showed that the protective association was primarily driven by non-PACG glaucoma (89.7% of cases), with significant effects at the ≥ 300 min/week threshold (HR = 0.83, 95% CI: 0.72–0.95, *P* = 0.008). Of note, 65.7% of cases were coded as other or unspecified glaucoma. Sex-stratified analyses for non-PACG glaucoma revealed the protective effect was particularly strong in men (≥ 300 min/week: HR = 0.73, 95% CI: 0.60–0.88, *P* = 0.001), but not significant in women (HR = 0.98, 95% CI: 0.79–1.21, *P* = 0.845). Associations for POAG (23.9% of cases) and PACG (10.3% of cases) were not statistically significant, likely to reflect limited statistical power and distinct pathophysiological mechanisms, respectively (Table S4). A nominally significant interaction was detected between physical activity patterns and sex for glaucoma risk (*P* = 0.026). Given the multiple interaction tests conducted, this finding should be considered hypothesis-generating rather than confirmatory. The WW pattern was associated with reduced glaucoma risk exclusively in men at both the ≥ 150 min/week (HR = 0.81, 95% CI: 0.68–0.97, *P* = 0.024) and ≥ 300 min/week (HR = 0.75, 95% CI: 0.62–0.89, *P* = 0.001) thresholds.

The results also remained stable after implementing a 3-year washout period to mitigate potential reverse causality (Figures S4–S5). Furthermore, the associations remained consistent when using alternative concentration thresholds for defining the WW pattern (≥ 40% and ≥ 60% of weekly MVPA within 1–2 days), confirming the robustness of our primary classification (Table S5).

Fine-Gray subdistribution hazard analyses treating death as a competing event (n = 3,411 deaths, 4.0% of cohort) yielded results consistent with primary Cox analyses (Table S6). The protective associations of the WW pattern remained significant for cataract (subdistribution hazard ratio [SHR] = 0.90, 95% CI: 0.86–0.95, *P* < 0.001) and AMD (SHR = 0.87, 95% CI: 0.77–0.98, *P* = 0.027), while the association with DR became marginally non-significant (SHR = 0.76, 95% CI: 0.56–1.02, *P* = 0.065).

## Discussion

Physical activity has emerged as a modifiable factor in preventing age-related diseases, yet the optimal temporal distribution of activity for ocular health has remained unexplored. While traditional guidelines emphasize regular daily exercise, contemporary lifestyle constraints have popularized the WW pattern, which concentrates weekly physical activity into one or two sessions. Using objective accelerometer data from a large prospective cohort, our study provides the first comprehensive evidence that this concentrated activity pattern is associated with reduced risks of multiple age-related eye diseases. When directly compared using the regularly active pattern as reference, the protective associations were statistically comparable for most outcomes, indicating that the WW pattern represents a viable alternative rather than an inferior approach to disease prevention.

Our findings revealed that the WW pattern was associated with reduced risks of cataract, DR, and AMD at the minimum activity threshold recommended by the World Health Organization [9, 22]. At higher activity levels, the protective effect of the WW pattern persisted for cataract and showed a sex-specific association with glaucoma, primarily driven by non-PACG subtypes including POAG. This finding extends previous cardiovascular, metabolic, and neurological research to the domain of ocular health [11–13, 15]. Restricted cubic spline analyses further revealed disease-specific dose–response patterns, with significant non-linear associations observed for cataract and AMD but not for DR and glaucoma. These distinct patterns suggest that optimal physical activity thresholds may vary by specific ocular pathology, potentially reflecting differences in underlying pathophysiological susceptibilities to exercise-induced adaptations. A key strength of our study lies in the objective measurement of physical activity through accelerometry, which overcomes the substantial misclassification inherent in self-reported assessments. This methodological advantage is evidenced by our finding that WW participants comprised a substantially larger proportion of the cohort compared to previous self-report-based studies [29], a proportion corroborated by independent accelerometer data from the National Health and Nutrition Examination Survey [30]. This disparity suggests that WW behavior is far more prevalent and achievable than previously recognized, with important implications for public health messaging.

Several mechanisms may explain the broader protective associations observed with the WW pattern compared with the regularly active pattern, despite similar total MVPA volumes. First, the concentrated exercise sessions characteristic of the WW pattern may elicit more pronounced acute physiological responses. Longer, uninterrupted exercise bouts can sustain hemodynamic benefits, such as improved ocular perfusion, for extended periods and stimulate more robust antioxidant and anti-inflammatory signaling (e.g., via the Nrf2 pathway and myokine release) than shorter, fragmented bouts of daily activity [31–33]. Second, as wrist-worn accelerometry does not capture activity type, we hypothesize that WWs may engage in more purposeful outdoor leisure activities (e.g., hiking or sports) compared to the occupational or domestic MVPA often found in daily active patterns [34]. Outdoor activity increases sunlight exposure and vitamin D synthesis, both of which have been linked to reduced risks of AMD, DR, and cataract [35]. Third, the retina is an extension of the central nervous system. Emerging evidence suggests that concentrated physical activity may enhance neurotrophic factor expression (such as brain-derived neurotrophic factor), potentially offering a protective effect against neurodegenerative ocular conditions like glaucoma [14]. We emphasize that these mechanistic hypotheses require direct experimental validation, and our findings should be interpreted as a basis for future studies incorporating objective measures of retinal physiology and systemic biomarkers.

A nominally significant interaction was observed between physical activity patterns and sex for glaucoma risk (*P* = 0.026). Although this finding did not survive Bonferroni correction given the multiple interaction tests performed, the biological plausibility of sex-specific responses and the observed dose–response relationship at both activity thresholds warrant further consideration. Specifically, our observation of male-specific glaucoma protection with the WW pattern may reflect fundamental differences in hormonal regulation of intraocular pressure and optic nerve perfusion. In women, estrogen likely confers baseline neuroprotection through enhanced nitric oxide synthase activity [36], improved aqueous humor dynamics, and optimized ocular blood flow [36], potentially creating a ceiling effect that diminishes incremental benefits from physical activity. Conversely, men lacking this hormonal protection may exhibit greater susceptibility to sympathetic activation and vascular reactivity, thereby potentially deriving enhanced ocular benefits from activity-induced reductions in intraocular pressure and improved optic nerve perfusion [7]. The predominance of non-PACG subtypes in driving these protective associations suggests that physical activity patterns may preferentially influence pressure-dependent mechanisms. This finding contrasts with previous UK Biobank research showing no association between total physical activity volume and glaucoma [37], suggesting that temporal distribution rather than cumulative volume may be more relevant for activating these sex-specific protective pathways. Nevertheless, given the exploratory nature of this interaction analysis, these results should be interpreted cautiously and require replication in independent cohorts.

The disease-specific variations in protective associations at higher activity thresholds suggest distinct pathophysiological susceptibilities to exercise-induced metabolic and vascular adaptations. Cataract and glaucoma may be more responsive to sustained metabolic improvements and pressure regulation achievable through concentrated sessions, whereas DR and AMD may exhibit a non-linear dose–response relationship. Supporting this, the attenuation of protective associations for DR and AMD at the ≥ 300 min/week threshold aligns with findings from a Korean cohort study, which reported that vigorous physical activity was paradoxically associated with increased risk of neovascular AMD, particularly among those exercising ≥ 5 times per week [38]. Mechanistically, the choroid is highly sensitive to hemodynamic changes, with thickness varying with body position; high-volume concentrated exercise may therefore induce acute fluctuations in choroidal circulation that could promote pathological angiogenesis [39]. Additionally, the smaller sample size at the ≥ 300 min/week threshold (n = 18,336 vs. 37,486) may have reduced statistical power. However, epidemiologic studies cannot establish causal mechanisms, and experimental studies are warranted to elucidate these relationships.

These findings carry important implications for individuals facing temporal or logistical barriers to daily exercise. The WW pattern offers a flexible, pragmatic alternative for preventing age-related eye diseases among those with demanding work schedules, caregiving responsibilities, or limited access to exercise facilities. However, this concentrated approach is not without risks. Previous research has documented elevated risks of musculoskeletal injuries and certain dermatologic conditions associated with sporadic high-intensity activity [40, 41], particularly among untrained individuals. Therefore, clinical recommendations should emphasize gradual progression, appropriate warm-up protocols, and medical consultation prior to adopting concentrated activity patterns, especially for older adults with pre-existing conditions.

Several limitations warrant consideration when interpreting our findings. First, physical activity was assessed via a single 7-day accelerometer measurement, which may not fully reflect longer-term behavioral changes. However, previous validation studies in the UK Biobank and similar cohorts have demonstrated good reproducibility, with intraclass correlation coefficients ranging from 0.67 to 0.82 over 2–4 years [42, 43] and strong correlations with activity levels observed over nearly 4 years [44]. While a single measurement adequately captures stable behavioral patterns, future studies incorporating longer or repeated measurements are warranted to confirm our findings. Second, despite excluding individuals developing outcomes within 0.5 or 3 years post-baseline to minimize reverse causality, we cannot entirely rule out that subclinical disease may have influenced baseline activity levels. Third, the current study utilized daily-level summary statistics provided by the UK Biobank rather than bout-level intensity metrics derived from raw 100 Hz data. As a result, we could not distinguish whether the broader protective associations of the WW pattern are primarily attributable to the temporal concentration of activity or to higher peak intensities during those concentrated bouts. While literature suggests that WWs may engage in more purposeful, high-intensity exercise compared to the incidental activity typical of regular patterns [45], further research using raw accelerometry is needed to isolate these factors. Fourth, measurement of certain covariates at baseline rather than concurrent with accelerometry introduces potential temporal misalignment, though the stability of most measured factors over short periods likely minimizes this concern. Fifth, we applied a Bonferroni correction to account for multiple hypothesis testing given the number of outcomes and thresholds analyzed. The protective association between the WW pattern and cataract remained significant after this adjustment (*P* < 0.0031). However, the low incidence of DR (n = 280 events; 0.41 per 1,000 person-years) yielded wide confidence intervals, particularly within the WW subgroup (n = 75; HR = 0.74, 95% CI: 0.55–0.99). Although point estimates suggest a protective effect, the limited events reduce precision, and these findings require replication in cohorts with higher disease burden or longer follow-up. Other associations, while showing consistent protective trends, did not reach the adjusted significance threshold and should be considered exploratory pending independent replication. Finally, residual confounding from unmeasured factors such as dietary patterns, genetic susceptibility, and occupational exposures cannot be excluded despite comprehensive covariate adjustment. Notably, participants in the WW group were generally younger and healthier at baseline. Although statistical adjustment attenuates this healthy user bias, it may not eliminate it entirely, and results should be interpreted within this context.

Future research should prioritize several key questions to advance our understanding of physical activity patterns and ocular health. First, sex-stratified studies incorporating hormonal profiling and intraocular pressure monitoring during and between WW activity sessions would elucidate the mechanistic basis for male-specific glaucoma protection. Second, comparative analyses of acute versus chronic physiological responses to concentrated versus distributed activity would clarify disease-specific optimal temporal patterns. Third, intervention trials randomizing participants to WW versus regular activity schedules while monitoring biomarkers of inflammation, oxidative stress, glucose metabolism, and ocular hemodynamics would establish causality and identify modifiable intermediary pathways. Fourth, investigations in diverse populations with varying baseline disease prevalence and genetic backgrounds would determine the generalizability of our findings. Finally, long-term studies examining whether WW patterns maintain protective associations over decades would inform sustainable lifestyle recommendations.

## Conclusion

Our study demonstrates that the WW physical activity pattern, characterized by concentrating MVPA into one or two days per week, is associated with significantly reduced risks of DR, cataract, and AMD. The WW pattern showed broader protective associations compared to regular activity, although direct comparisons did not reach statistical significance. Notably, protection against glaucoma was observed only among male participants. The underlying mechanisms warrant further investigation. These findings suggest that the WW approach represents a feasible and flexible alternative for individuals unable to exercise daily, offering valuable insights for physical activity guidelines and clinical recommendations.

## Supplementary Information

Supplementary Material 1.

## Data Availability

The UK Biobank Resource (application 302523) provided access to data utilized in this study. Privacy regulations prevent the sharing of these datasets with additional researchers. Access to UK Biobank datasets requires submission of applications via official pathways.
