# Supplementary material for: Accelerometer-derived “weekend warrior” physical activity pattern and risk of age-related eye diseases: a prospective cohort study
Source: Eye Vis (Lond). 2026 Mar 10;13:11. doi: 10.1186/s40662-026-00480-6 (PMC12973921; doi:10.1186/s40662-026-00480-6)

**Supplementary Materials**

**Table S1.** Ascertainment of age-related eye diseases in the UK Biobank.

| **Diseases** | **ICD-10** |
| --- | --- |
| Cataract | H25.0, H25.1, H25.2, H25.8, H25.9, H26.0, H26.2, H26.3, H26.4, H26.8, H26.9, H28.0, H28.1, H28.2 |
| Diabetic retinopathy | H36.0 |
| Age-related macular degeneration | H35.3 |
| Glaucoma | H40.0, H40.1, H40.2, H40.8, H40.9 |

ICD = International Classification of Diseases.

**Table S2.** The definitions of covariates in the UK Biobank.

| **Covariates** | **Definitions** | **Field ID** |
| --- | --- | --- |
| Age | Age at recruitment, date of birth. | 21002, 33 |
| Sex | Male and female. | 31 |
| Ethnicity | White, Asian, Black, Other. “Chinese” included in category Asian, and “Mixed” included in category Other. | 21000 |
| Educational attainment | Age completed full time education, qualifications. | 845,  6138 |
| Townsend deprivation index | Townsend deprivation index calculated immediately prior to participant joining UK Biobank. Based on the preceding national census output areas. Each participant is assigned a score corresponding to the output area in which their postcode is located. | 22189 |
| BMI | BMI was constructed from height and weight measured during the initial Assessment Centre visit. | 21001 |
| Alcohol drinker status | Alcohol drinker status was defined as three groups: Current, Previous, Never. | 20117 |
| Smoking status | Smoking status was defined as three groups: Current, Previous, Never. | 20116 |
| Hypertension | Touchscreen questionnaire and verbal interview: “self-reported hypertension or anti-hypertensive medication use.” | 6150, 20002, 6177, 6153 |
| Diabetes | Touchscreen questionnaire and verbal interview: “self-reported diabetes (diabetes, type 1 diabetes or type 2 diabetes) or insulin use.” | 2443, 20002, 6177, 6153 |
| Employment | In paid employment or self-employed coded as Paid. | 6142 |

BMI = body mass index

**Table S3.** Baseline characteristics comparison between imputed and non-imputed data groups.

| **Variable** | **Imputed**  **(n = 92,563)** | **Non-imputed**  **(n = 90,924)** | **Total**  **(n = 183,487)** | ***P* value** |
| --- | --- | --- | --- | --- |
| Age (years) | 63.0 [55.8; 68.1] | 63.0 [55.8; 68.1] | 63.0 [55.8; 68.1] | 0.841 |
| Sex, n (%) |  |  | - | 0.931 |
| Female | 52,191 (56.4%) | 51,286 (56.4%) | 103,477 (56.4%) |  |
| Male | 40,372 (43.6%) | 39,638 (43.6%) | 80,010 (43.6%) |  |
| Ethnicity, n (%) |  |  | - | 0.427 |
| White | 89,658 (96.9%) | 88,178 (97.0%) | 177,836 (96.9%) |  |
| Black | 788 (0.9%) | 754 (0.8%) | 1,542 (0.8%) |  |
| Asian | 1,068 (1.2%) | 1,028 (1.1%) | 2,096 (1.1%) |  |
| Other | 1,049 (1.1%) | 964 (1.1%) | 2,013 (1.1%) |  |
| Townsend index | −2.4 [−3.8; −0.2] | −2.4 [−3.8; −0.2] | −2.4 [−3.8; −0.2] | 0.442 |
| Education (years) | 20.0 [16.0; 20.0] | 20.0 [16.0;20.0] | 20.0 [16.0;20.0] | 0.859 |
| Employment, n (%) |  |  | - | 0.946 |
| Retired/unemployed | 36,600 (39.5%) | 35,937 (39.5%) | 72,537 (39.5%) |  |
| Employed | 55,963 (60.5%) | 54,987 (60.5%) | 110,950 (60.5%) |  |
| Alcohol drinking, n (%) |  |  | - | 0.892 |
| Never | 2,733 (3.0%) | 2,655 (2.9%) | 5,388 (2.9%) |  |
| Previous | 2,555 (2.8%) | 2,494 (2.7%) | 5,049 (2.8%) |  |
| Current | 87,275 (94.3%) | 85,775 (94.3%) | 173,050 (94.3%) |  |
| BMI (kg/m²) | 26.0 [23.6;29.0] | 26.0 [23.6;29.0] | 26.0 [23.6;29.0] | 0.846 |
| Smoking, n (%) |  |  | - | 0.953 |
| Never | 52,925 (57.2%) | 51,993 (57.2%) | 104,918 (57.2%) |  |
| Previous | 33,424 (36.1%) | 32,859 (36.1%) | 66,283 (36.1%) |  |
| Current | 6,214 (6.7%) | 6,072 (6.7%) | 12,286 (6.7%) |  |
| Hypertension, n (%) |  |  | - | 0.933 |
| No | 69,491 (75.1%) | 68,277 (75.1%) | 137,768 (75.1%) |  |
| Yes | 23,072 (24.9%) | 22,647 (24.9%) | 45,719 (24.9%) |  |
| Diabetes, n (%) |  |  | - | 0.971 |
| No | 89,226 (96.4%) | 87,650 (96.4%) | 176,876 (96.4%) |  |
| Yes | 3,337 (3.6%) | 3,274 (3.6%) | 6,611 (3.6%) |  |
| Glaucoma, n (%) |  |  | - | 0.697 |
| No | 89,931 (97.2%) | 88,367 (97.2%) | 178,298 (97.2%) |  |
| Yes | 2,632 (2.8%) | 2,557 (2.8%) | 5,189 (2.8%) |  |
| Cataract, n (%) |  |  | - | 0.802 |
| No | 80,842 (87.3%) | 79,447 (87.4%) | 160,289 (87.4%) |  |
| Yes | 11,721 (12.7%) | 11,477 (12.6%) | 23,198 (12.6%) |  |
| Diabetic retinopathy, n (%) |  |  | - | 0.904 |
| No | 92,090 (99.5%) | 90,464 (99.5%) | 182,554 (99.5%) |  |
| Yes | 473 (0.5%) | 460 (0.5%) | 933 (0.5%) |  |
| Age-related macular degeneration, n (%) |  |  | - | 0.900 |
| No | 90,620 (97.9%) | 89,024 (97.9%) | 179,644 (97.9%) |  |
| Yes | 1,943 (2.1%) | 1,900 (2.1%) | 3,843 (2.1%) |  |

BMI = body mass index

**Table S4.** Association between physical activity patterns and glaucoma risk by subtype and sex.

| **Outcome** | **Activity pattern** | **N events** | **HR (95% CI)** | ***P* value** |
| --- | --- | --- | --- | --- |
| ***150 min/week threshold*** | | | | |
| **Total glaucoma** | Inactive | 572 | 1.00 (reference) | – |
|  | Regular | 371 | 1.07 (0.93–1.22) | 0.359 |
|  | Weekend warrior | 653 | 0.93 (0.83–1.04) | 0.220 |
| **POAG (H40.1)** | Inactive | 125 | 1.00 (reference) | – |
|  | Regular | 90 | 1.15 (0.87–1.53) | 0.322 |
|  | Weekend warrior | 167 | 1.05 (0.83–1.34) | 0.686 |
| **PACG (H40.2)** | Inactive | 56 | 1.00 (reference) | – |
|  | Regular | 38 | 1.19 (0.77–1.82) | 0.438 |
|  | Weekend warrior | 71 | 1.11 (0.77–1.60) | 0.569 |
| **Non-PACG** | Inactive | 516 | 1.00 (reference) | – |
|  | Regular | 333 | 1.05 (0.91–1.22) | 0.477 |
|  | Weekend warrior | 582 | 0.91 (0.81–1.03) | 0.138 |
| **Non-PACG (men)** | Inactive | 211 | 1.00 (reference) | – |
|  | Regular | 194 | 1.04 (0.85–1.27) | 0.734 |
|  | Weekend warrior | 302 | **0.80 (0.67–0.96)** | **0.017** |
| **Non-PACG (women)** | Inactive | 305 | 1.00 (reference) | – |
|  | Regular | 139 | 1.03 (0.84–1.27) | 0.756 |
|  | Weekend warrior | 280 | 1.03 (0.87–1.21) | 0.764 |
| ***300 min/week threshold*** | | | | |
| **Total glaucoma** | Inactive | 1024 | 1.00 (reference) | – |
|  | Regular | 276 | 1.03 (0.90–1.18) | 0.702 |
|  | Weekend warrior | 296 | **0.85 (0.75–0.97)** | **0.019** |
| **POAG (H40.1)** | Inactive | 236 | 1.00 (reference) | – |
|  | Regular | 68 | 1.08 (0.82–1.43) | 0.588 |
|  | Weekend warrior | 78 | 0.94 (0.73–1.23) | 0.673 |
| **PACG (H40.2)** | Inactive | 100 | 1.00 (reference) | – |
|  | Regular | 30 | 1.20 (0.79–1.84) | 0.387 |
|  | Weekend warrior | 35 | 1.12 (0.75–1.67) | 0.582 |
| **Non-PACG** | Inactive | 924 | 1.00 (reference) | – |
|  | Regular | 246 | 1.01 (0.87–1.17) | 0.904 |
|  | Weekend warrior | 261 | **0.83 (0.72–0.95)** | **0.008** |
| **Non-PACG (men)** | Inactive | 406 | 1.00 (reference) | – |
|  | Regular | 151 | 1.01 (0.83–1.22) | 0.939 |
|  | Weekend warrior | 150 | **0.73 (0.60–0.88)** | **0.001** |
| **Non-PACG (women)** | Inactive | 518 | 1.00 (reference) | – |
|  | Regular | 95 | 0.98 (0.79–1.23) | 0.890 |
|  | Weekend warrior | 111 | 0.98 (0.79–1.21) | 0.845 |

POAG = primary open-angle glaucoma; PACG = primary angle-closure glaucoma; HR = hazard ratio; CI = confidence interval

All models were adjusted for age, sex (except sex-stratified analyses), Townsend deprivation index, ethnicity, education, employment status, smoking status, alcohol consumption, body mass index, hypertension, and diabetes. Bold values indicate statistical significance (*P* < 0.05).

**Table S5.** Sensitivity analyses of weekend warrior pattern associations using alternative concentration thresholds.

| **Outcome** | **≥ 40% threshold** | |  | **≥ 50% threshold** | | |  | | **≥ 60% threshold** | | |
| --- | --- | --- | --- | --- | --- | --- | --- | --- | --- | --- | --- |
|  | **HR (95% CI)** | ***P*** | |  | **HR (95% CI)** | ***P*** |  | **HR (95% CI)** | | ***P*** |  |
| Cataract | 0.90 (0.86–0.95) | < 0.001 | |  | 0.89 (0.84–0.94) | < 0.001 |  | 0.91 (0.85–0.96) | | 0.002 |  |
| Diabetic retinopathy | 0.81 (0.63–1.06) | 0.120 | |  | 0.74 (0.55–0.99) | 0.041 |  | 0.62 (0.42–0.91) | | 0.015 |  |
| Age-related macular degeneration | 0.87 (0.77–0.98) | 0.017 | |  | 0.85 (0.75–0.97) | 0.013 |  | 0.87 (0.76–1.01) | | 0.073 |  |
| Glaucoma | 0.96 (0.86–1.07) | 0.495 | |  | 0.93 (0.83–1.04) | 0.220 |  | 0.90 (0.79–1.03) | | 0.141 |  |

HR = hazard ratio; CI = confidence interval; WW = weekend warrior; MVPA = moderate-to-vigorous physical activity

All models were adjusted for age, sex, Townsend deprivation index, ethnicity, education, employment status, smoking status, alcohol consumption, body mass index, hypertension, and diabetes. WW was defined as accumulating the specified percentage of total weekly MVPA within 1–2 days among participants meeting the ≥ 150 min/week threshold.

**Table S6.** Fine-Gray subdistribution hazard ratios for age-related eye diseases with death as a competing risk.

| **Outcome** | **Activity pattern** | **N events** | **Competing events** | **Censored** | **SHR (95% CI)** |
| --- | --- | --- | --- | --- | --- |
| **Cataract** | Inactive | 2,927 | 1,135 | 24,701 | 1.00 (reference) |
|  | Regular | 1,432 | 637 | 17,953 | 0.94 (0.88–1.00) |
|  | Weekend warrior | 3,034 | 1,323 | 33,129 | 0.90 (0.86–0.95) |
| **Diabetic retinopathy** | Inactive | 109 | 1,252 | 27,402 | 1.00 (reference) |
|  | Regular | 62 | 654 | 19,306 | 1.00 (0.72–1.39) |
|  | Weekend warrior | 109 | 1,467 | 35,910 | 0.76 (0.56–1.02) |
| **Age-related macular degeneration** | Inactive | 536 | 1,228 | 26,999 | 1.00 (reference) |
|  | Regular | 290 | 655 | 19,077 | 0.91 (0.78–1.06) |
|  | Weekend warrior | 505 | 1,469 | 35,512 | 0.87 (0.77–0.98) |
| **Glaucoma** | Inactive | 509 | 1,204 | 27,050 | 1.00 (reference) |
|  | Regular | 366 | 649 | 19,007 | 1.08 (0.94–1.23) |
|  | Weekend warrior | 721 | 1,465 | 35,300 | 0.94 (0.84–1.06) |

SHR = subdistribution hazard ratio; CI = confidence interval; MVPA = moderate-to-vigorous physical activity

Competing events refer to deaths that occurred before the diagnosis of the eye disease outcome (n = 3,411 total deaths, 4.0% of cohort). Models were adjusted for age, sex, Townsend deprivation index, ethnicity, education, employment status, smoking status, alcohol consumption, body mass index, hypertension, and diabetes. Physical activity threshold: ≥ 150 minutes/week of moderate-to-vigorous physical activity.

**Figure S1.** Dose-response relationships between weekly moderate-to-vigorous physical activity and risk of age-related eye diseases. Hazard ratios (HRs) estimated using restricted cubic splines (3 degrees of freedom) with 150 min/week as reference (HR = 1.0). Shaded areas: 95% confidence intervals. Light red region: inactive group (< 150 min/week). Adjusted for age, sex, Townsend deprivation index, ethnicity, education, employment, smoking, alcohol consumption, body mass index, hypertension, and diabetes. AMD, age-related macular degeneration; DR, diabetic retinopathy; MVPA, moderate to vigorous physical activity

**

**

**Figure S2.** Associations between physical activity pattern (≥ 150 min/week) and incidence of age-related eye diseases following data imputation. Participants were categorized as weekend warriors (WW), active regular, or inactive (reference group). AMD, age-related macular degeneration; DR, diabetic retinopathy; HR, hazard ratio; CI, confidence interval. Models were adjusted for age, sex, Townsend deprivation index, ethnicity, education, employment status, smoking status, alcohol consumption, body mass index, hypertension, and diabetes.


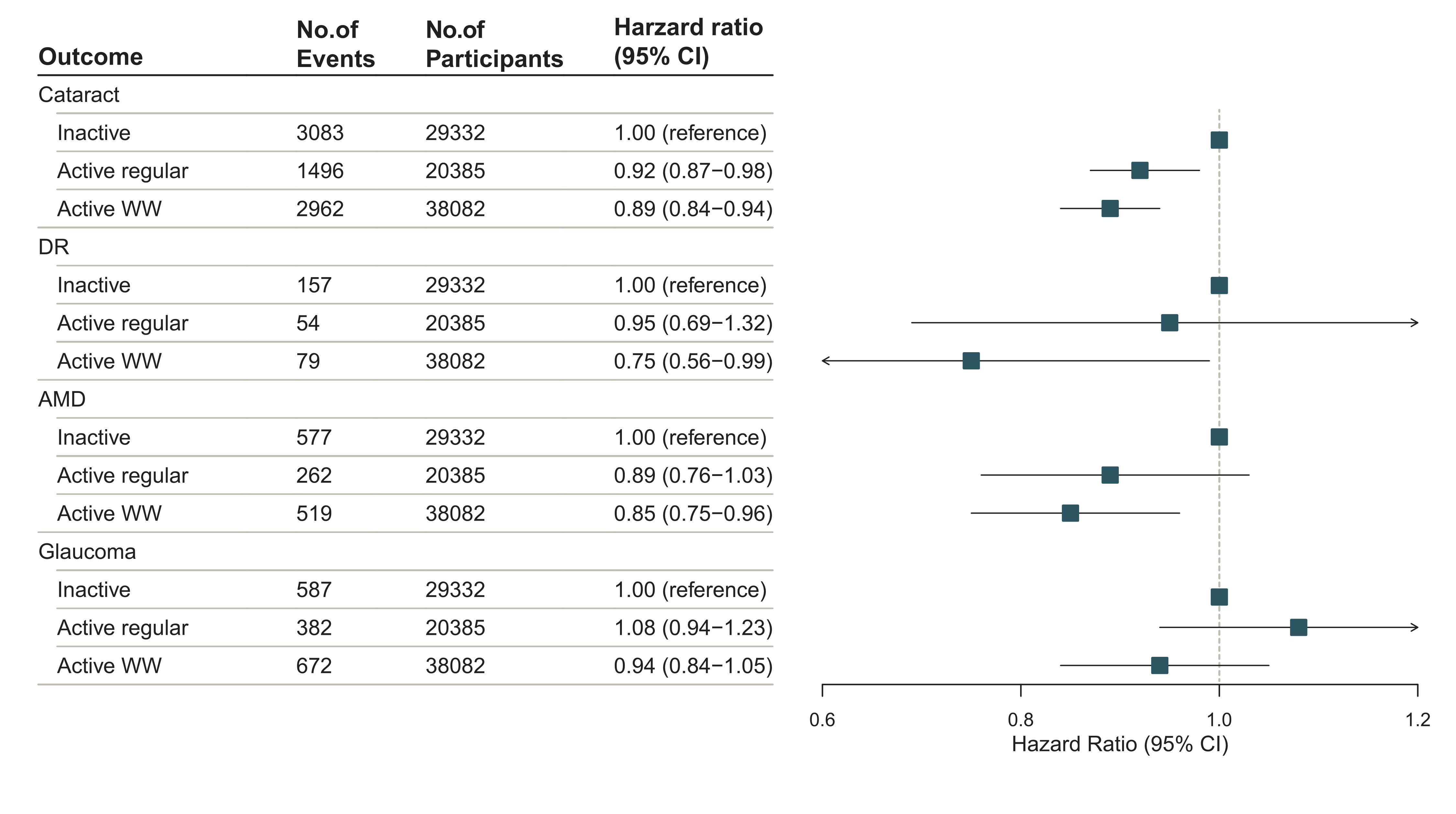


**Figure S3.** Associations between physical activity pattern (≥ 300 min/week) and incidence of age-related eye diseases following data imputation. Participants were categorized as weekend warriors (WW), active regular, or inactive (reference group). AMD, age-related macular degeneration; DR, diabetic retinopathy; HR, hazard ratio; CI, confidence interval. Models were adjusted for age, sex, Townsend deprivation index, ethnicity, education, employment status, smoking status, alcohol consumption, body mass index, hypertension, and diabetes.


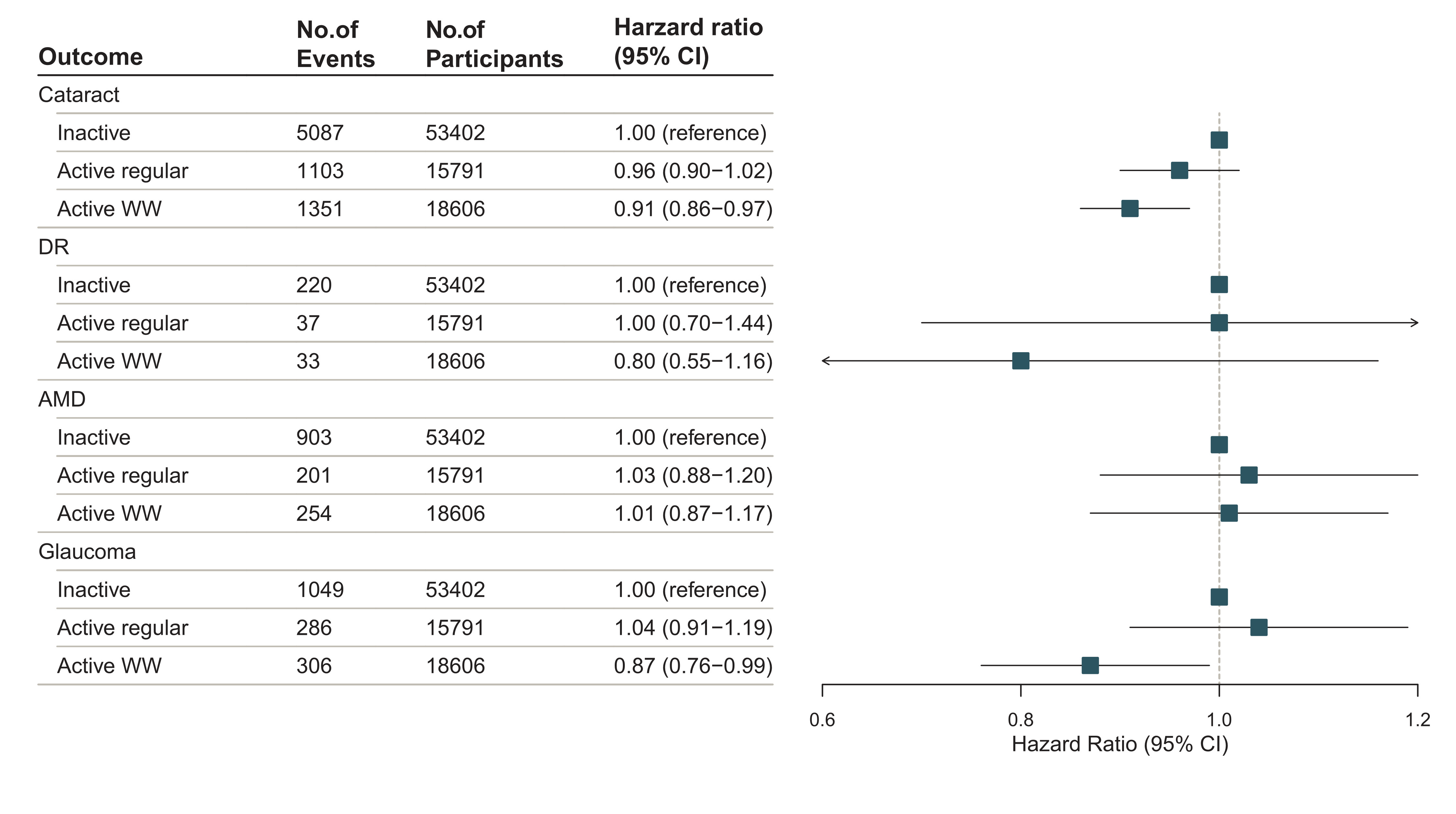


**Figure S4.** Associations between physical activity pattern (≥ 150 min/week) and incidence of age-related eye diseases after excluding cases with onset during the initial 3 years. Participants were categorized as weekend warriors (WW), active regular, or inactive (reference group). AMD, age-related macular degeneration; DR, diabetic retinopathy; HR, hazard ratio; CI, confidence interval. Models were adjusted for age, sex, Townsend deprivation index, ethnicity, education, employment status, smoking status, alcohol consumption, body mass index, hypertension, and diabetes.


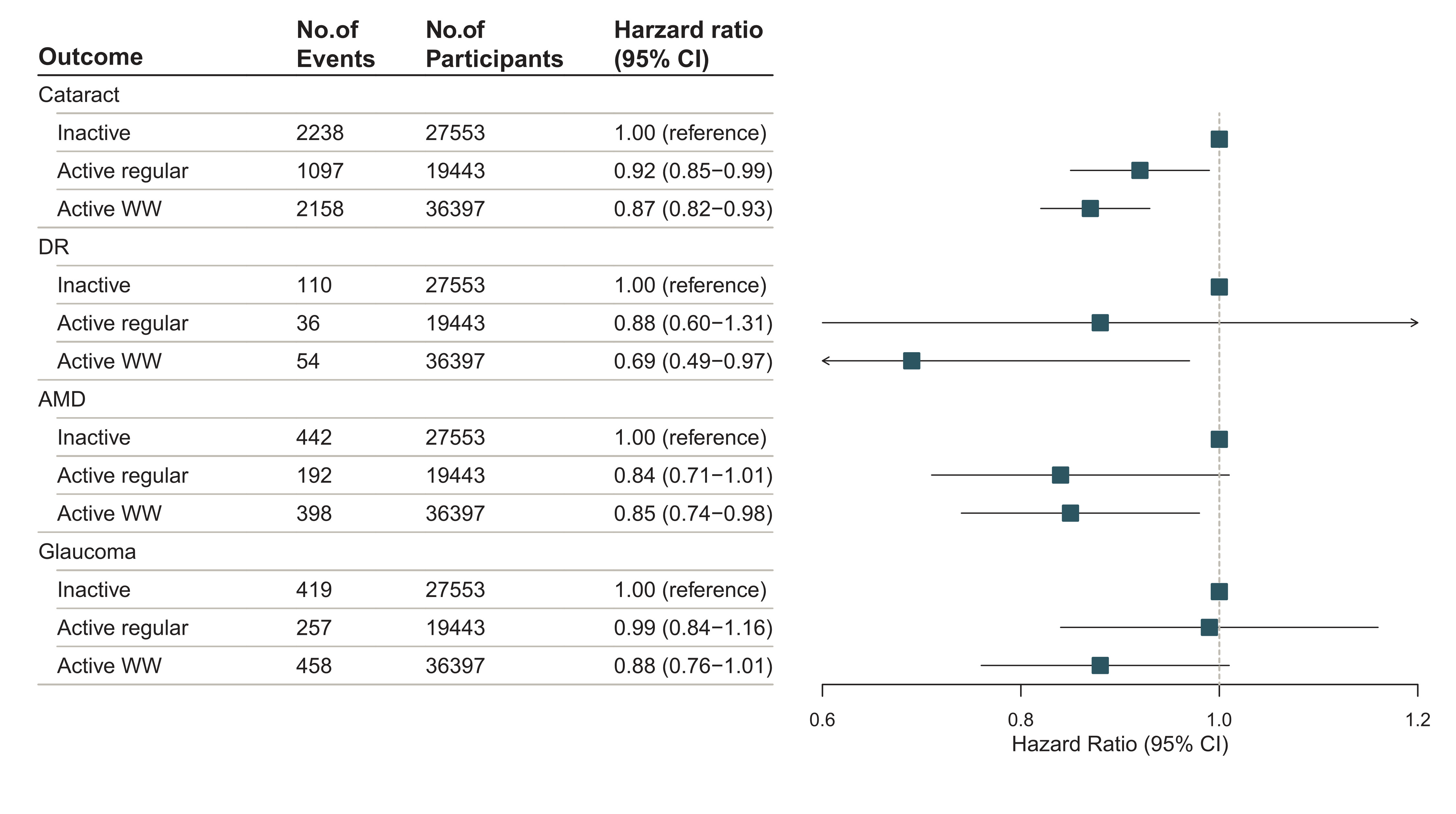


**Figure S5.** Associations between physical activity pattern (≥ 300 min/week) and incidence of age-related eye diseases after excluding cases with onset during the initial 3 years. Participants were categorized as weekend warriors (WW), active regular, or inactive (reference group). AMD, age-related macular degeneration; DR, diabetic retinopathy; HR, hazard ratio; CI, confidence interval. Models were adjusted for age, sex, Townsend deprivation index, ethnicity, education, employment status, smoking status, alcohol consumption, body mass index, hypertension, and diabetes.


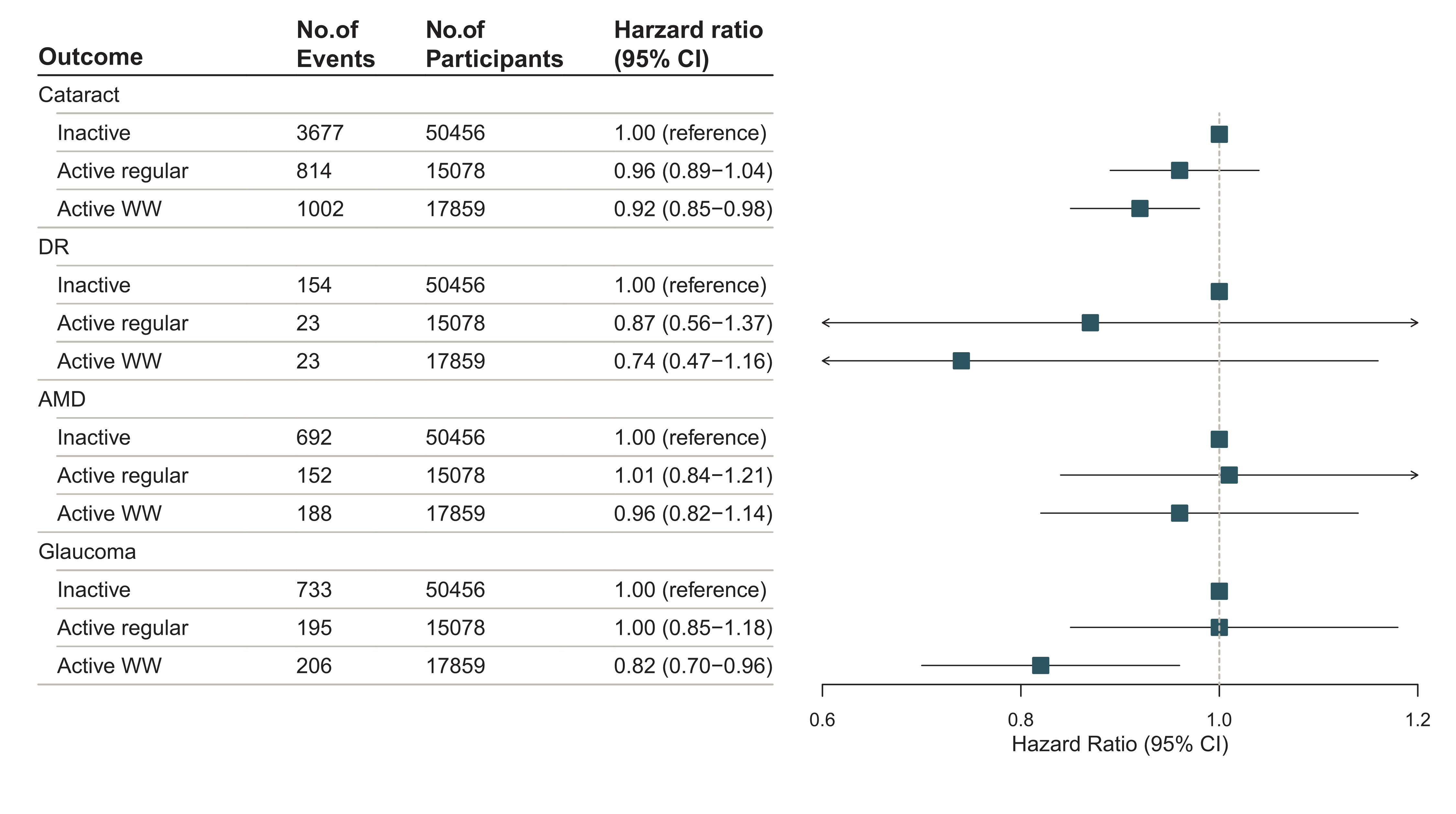

Supplement: Supplementary file 1 — Supplementary Material 1. [file 40662_2026_480_MOESM1_ESM.docx]
